# Corticotropin-Releasing Hormone Receptor Alters the Tumor Development and Growth in *Apcmin/+* Mice and in a Chemically-Induced Model of Colon Cancer

**DOI:** 10.3390/ijms22031043

**Published:** 2021-01-21

**Authors:** Yunna Lee, Elise L. Ma, Marisa Patel, Gayoung Kim, Cody Howe, Charalabos Pothoulakis, Yong Sung Kim, Eunok Im, Sang Hoon Rhee

**Affiliations:** 1 College of Pharmacy, Pusan National University, Busan 46241, Korea; yunnalee@pusan.ac.kr; 2 Inflammatory Bowel Disease Center, and Center for Systems Biomedicine, Vatcher and Tamar Manoukian Division of Digestive Diseases, David Geffen School of Medicine, UCLA, Los Angeles, CA 90095, USA; elma@mednet.ucla.edu (E.L.M.); cpothoulakis@mednet.ucla.edu (C.P.); 3 Department of Biological Sciences, Oakland University, Rochester, MI 48309, USA; marisapatel@oakland.edu (M.P.); gayoungkim@oakland.edu (G.K.); cshowe@oakland.edu (C.H.); 4 Digestive Disease Research Institute and GutnFood Healthcare Inc., School of Medicine, Wonkwang University, Iksan 54538, Korea; wms89@hanmail.net

**Keywords:** cyclooxygenase 2, neuropeptide, stress, tumorigenicity, gastrointestinal, colitis, azoxymethane

## Abstract

The neuroendocrine circuit of the corticotropin-releasing hormone (CRH) family peptides, via their cognate receptors CRHR1 and CRHR2, copes with psychological stress. However, peripheral effects of the CRH system in colon cancer remains elusive. Thus, we investigate the role of CRHR1 and CRHR2 in colon cancer. Human colon cancer biopsies were used to measure the mRNA levels of the *CRH* family by quantitative real-time PCR. Two animal models of colon cancer were used: *Apcmin/+* mice and azoxymethane (AOM)/dextran sulfate sodium (DSS)-treated mice. The mRNA levels of *CRHR2* and *UCN III* are reduced in human colon cancer tissues compared to those of normal tissues. *Crhr1* deletion suppresses the tumor development and growth in *Apcmin/+* mice, while *Crhr2* deficiency exacerbates the tumorigenicity. *Crhr1* deficiency not only inhibits the expression of tumor-promoting cyclooxygenase 2, but also upregulates tumor-suppressing phospholipase A2 in *Apcmin/+* mice; however, *Crhr2* deficiency does not change these expressions. In the AOM/DSS model, *Crhr2* deficiency worsens the tumorigenesis. In conclusion, *Crhr1* deficiency confers tumor-suppressing effects in *Apcmin/+* mice, but *Crhr2* deficiency worsens the tumorigenicity in both *Apcmin/+* and AOM/DSS-treated mice. Therefore, pharmacological inhibitors of CRHR1 or activators of CRHR2 could be of significance as anti-colon cancer drugs.

## 1. Introduction

Corticotropin-releasing hormone (CRH, also known as corticotrophin-releasing factor) is a group of hypothalamic peptides that regulates the psychological responses of the hypothalamic-pituitary-adrenal (HPA) axis [1,2]. CRH is secreted from the hypothalamus in response to stress, and subsequently induces the production of stress hormones such as glucocorticoids and adrenocorticotropin [3]. CRH-induced glucocorticoids mitigate the stress response by suppressing the endocrine activity of the hypothalamus and the pituitary gland [4]. Given the ability of glucocorticoids to suppress inflammation and immune responses, the CRH family peptides additionally regulate the inflammation and immune responses at peripheral organs [5,6]. The CRH family peptides include CRH [7], urocortin I (UCN I) [8], UCN II (stresscopin-related peptide) [9], and UCN III (stresscopin) [10]. Each member acts via two distinct G protein-coupled receptors, CRH receptor 1 (CRHR1) and CRHR2 [3]. CRHR1 is mainly expressed in the brain. Accordingly, it is generally accepted that CRHR1 copes with stress responses of the HPA axis by inducing adrenal corticotropic hormone [11]. In contrast, CRHR2 is abundantly expressed in several peripheral organs, which were examined with rodents and humans [12]. CRHR2 is involved in regulating various cellular events, including mucosal repair in colitis [13], angiogenesis [14], and Fas-mediated apoptosis in colon cancer [15].

Regarding the receptor binding affinity, CRH and UCN I bind to both CRHR1 and CRHR2 in order to mediate their physiological and cellular effects [11]. It is worth noting that the binding affinity of CRH is highly biased toward CRHR1 [16]. UCN II and UCN III are the selective ligands for CRHR2 [9,10]. When the CRH family peptide binds to its cognate receptor, it activates the Gα subunit and subsequently the adenylyl cyclase/cAMP signaling pathway to mediate physiological and cellular responses. Through this signaling pathway, it is of significance that the CRH family also activates the gut neuroendocrine system to regulate the gut motility and inflammatory responses [2].

Previously, we have demonstrated that either loss of *Crhr1* encoding gene or a pharmacological blockade of CRHR1 confers protective effects in dextran sulfate sodium (DSS)-induced mouse colitis [14]. On the other hand, either *Crhr2* encoding gene deletion or CRHR2 inhibitor treatment exacerbates DSS-induced mouse colitis [14]. Loss of *Crhr1* encoding gene results in reduced microvascular density due to diminished expression of vascular endothelial growth factor A (VEGF-A) in the inflamed colon of mice. Indeed, CRHR1 stimulation with CRH activates AKT in microvascular endothelial cells, leading to enhanced formation of microvascular structures. Accordingly, the absence of CRHR1-mediated responses appears to suppress the angiogenesis in the intestine; thereby diminishing the inflammatory response in the gut. In contrast, *Crhr1* deficiency leads to increased angiogenesis with enhanced VEGF-A expression [14]. Therefore, blocking the CRHR2-mediated responses can enhance angiogenesis, thereby worsening intestinal inflammation [1].

Likewise, the CRH family peptides appear to play a pivotal role in the onset and progress of various pathological conditions in extra-hypothalamic peripheral organs [1,6]. Among them, the correlation of CRHRs with colon cancer has been poorly investigated. Therefore, in this study, we examine the impact of CRHRs on intestinal tumorigenesis using *Crhr1^−/−^* and *Crhr2^−/−^* mice that were tested in two experimental mouse models of colon cancer: the *Apcmin/+* and the azoxymethane (AOM)-DSS models. We found that the *Crhr1* deficiency confers the tumor-suppressing effect in the *Apcmin/+* mouse model, while the *Crhr2* deficiency exacerbates the tumorigenicity in these two experimental models. These data are clinically of significance in developing a prophylactic and therapeutic approach to colon cancer in humans.

## 2. Results

### 2.1. The mRNA Levels of Corticotropin-Releasing Hormone Receptor 2 (CRHR2) and Urocortin (UCN) III Are Reduced in Human Colon Cancer Tissues Compared to Those of Normal Tissues

**Figure 1:**
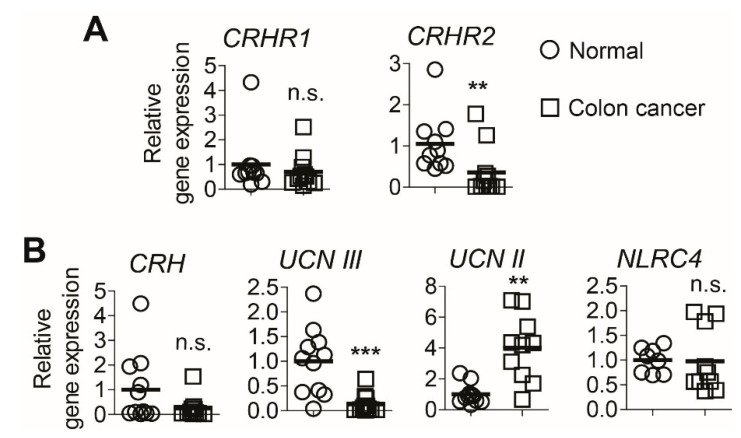
The mRNA levels of corticotropin-releasing hormone receptor 2 (*CRHR2)* and its cognate ligand urocortin (*UCN) III* are reduced in human colon cancer tissues relative to normal tissues. (**A**,**B**) Relative mRNA expression levels of *CRHR1* and *CRHR2* (**A**) and the *CRH* family member and *NLRC4* (**B**) were evaluated by quantitative real-time PCR (qPCR) with colon cancer tissues obtained from colon cancer patients (n = 11) and unmatched normal tissues (n = 10) independently obtained from healthy control subjects. ** *p* < 0.01, *** *p* < 0.001, n.s. stands for not significant (Mann–Whitney U test). Horizontal bar in each graph indicates mean.

Although the CRH family peptides mediate the neuroendocrine responses primarily in the nervous system, they are able to affect the onset and perpetuation of intestinal inflammation, indicating a role of the CRH family in maintaining intestinal homeostasis [14]. However, the impact of the CRH family on colon cancer has not been fully investigated. To address this issue, we first examined the mRNA expression levels of the *CRH* family and cognate receptors in human colon cancer tissues and unmatched normal colon tissues. We found that the mRNA level of *CRHR2* was substantially lower in colon cancer tissues than that of normal tissues, while the *CRHR1* levels are comparable between these tissues (Figure 1A).

Furthermore, the mRNA level of *UCN III*, a CRHR2-specific peptide among the CRH family, was dramatically reduced in colon cancer tissues compared to that of controls; however, the level of *CRH*, a CRHR1-restricted ligand, was not changed in these tissues (Figure 1B). Intriguingly, the level of CRHR2-targeting *UCN II* was markedly increased in colon cancer tissues compared to that of controls, while *NLRC4* levels are preserved. Given the reduced expression of *CRHR2* and its ligand *UCN III* in human colon cancer tissues, these findings indicate that the impairment of CRHR2-mediated responses should be associated with colon cancer development in humans, which may contribute to cancer metastasis or poor patient survival.

### 2.2. Loss of Crhr2 Gene Promotes Tumor Development in the Apcmin/+ Mouse, While Crhr1 Deficiency Suppresses the Tumorigenesis

**Figure 2:**
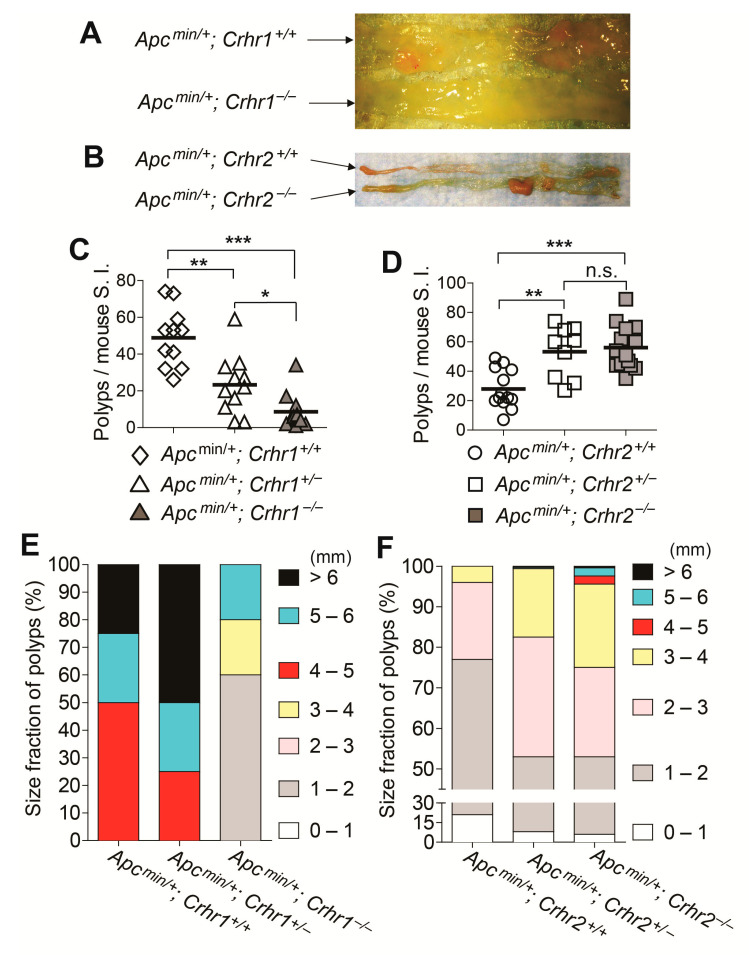
Loss of *Crhr1* gene reduces the intestinal tumor development and growth in the *Apcmin/+* background, while *Crhr2* gene deletion greatly increases the tumorigenesis. (**A**,**B**) Gross images of the tumors observed in the small intestine of *Apcmin/+; Crhr1^−/−^* mice and littermate *Apcmin/+; Crhr1^+/+^* mice, and *Apcmin/+; Crhr2^−/−^* mice and littermate *Apcmin/+; Crhr2^+/+^* mice at the age of 5 months. Presented are the representative images. (**C**,**D**) At 5 months old, the tissues were harvested. Using stereoscopic microscopy, we counted the number of tumors that were developed throughout the small intestine of each mouse. * *p* < 0.05, ** *p* < 0.01, *** *p* < 0.001 (Mann–Whitney U test). *Apcmin/+; Crhr1^+/+^* (n = 11), *Apcmin/+; Crhr1^+/−^* (n = 11), *Apcmin/+; Crhr1^−/−^* (n = 11), *Apcmin/+; Crhr2^+/+^* (n = 13), *Apcmin/+; Crhr2^+/−^* (n = 9), *Apcmin/+; Crhr2^−/−^* mice (n = 14). (**E**,**F**) Tumors observed throughout the small intestine were enumerated to analyze relative proportions (%) of tumor size. Adenomas were classified based on the tumor size: small (<2 mm diameter), medium-sized (2–4 mm), or large (>4 mm). The relative proportions of the size classes were counted and given as a percentage of the total number of adenomas.

To study the impact of CRHR-mediated responses in colon cancer, we utilized the *Apcmin/+* mouse model of intestinal epithelial tumorigenesis, in which tumor development occurs mainly in the small intestine [17,18]. Specifically, *Crhr1^−/−^* and *Crhr2^−/−^* mice were combined with *Apcmin/+* mice to generate *Apcmin/+; Crhr1^−/−^* mice and *Apcmin/+; Crhr2^−/−^* mice, respectively. Given the importance of mouse age in the tumorigenesis, we examined the tumor development of these mice at the age of 10 months. We observed massive development of tumor polyps throughout the small intestine of the mice (Figure 2A,B). The number of tumors developed in the small intestine of *Apcmin/+; Crhr1^−/−^* mice was substantially lower than that of littermate *Apcmin/+; Crhr1^+/+^* mice (Figure 2C). Likewise, the number of tumors was also reduced in the small intestine of *Apcmin/+; Crhr1^+/−^* mice compared to that of littermate *Apcmin/+; Crhr1^+/+^* mice. It is worth noting that the number of tumors developed in the intestine of *Apcmin/+; Crhr1^−/−^* mice is much lower than the tumors in *Apcmin/+; Crhr1^+/−^* mice. These data indicate that Crhr1 deficiency is capable of suppressing the tumor development in the *Apcmin/+* mouse model of colon cancer; the haplodeficiency of Crhr1 gene is also capable of suppressing the tumorigenesis.

In contrast, we found that both *Apcmin/+; Crhr2^−/−^* mice and *Apcmin/+; Crhr2^+/−^* mice had greatly increased numbers of tumors in their small intestine, compared to the tumor development in littermate *Apcmin/+; Crhr2^+/+^* mice (Figure 2D). However, the number of tumors between *Apcmin/+; Crhr2^−/−^* mice and *Apcmin/+; Crhr2^+/−^* mice were comparable. These data indicate that either Crhr2 gene deficiency or heterozygous deletion of *Crhr2* gene substantially increases the number of adenomas in the intestine of *Apcmin/+* mice.

Furthermore, we measured the adenoma size to examine the adenoma burden in the intestine. The percentage proportions of large adenoma (> 4 mm diameter) and medium-sized adenoma (2–4 mm) were greatly reduced in *Apcmin/+; Crhr1^−/−^* mice compared to those of littermate *Apcmin/+; Crhr1^+/−^* and *Apcmin/+; Crhr1^+/+^* mice (Figure 2E). Conversely, the percentage proportion of large adenomas was markedly increased in the intestine of *Apcmin/+; Crhr2^−/−^* mice compared to those of littermate *Apcmin/+; Crhr2^+/−^* and *Apcmin/+; Crhr2^+/+^* mice (Figure 2F). These data indicate that loss of *Crhr1* gene should suppress the tumor growth in the *Apcmin/+* mouse model, while *Crhr2* gene deficiency can enhance the tumor growth.

Taken together, our findings suggest that blocking CRHR1-mediated responses inhibit tumor development and growth in an *Apcmin/+* background, while inhibiting CRHR2-mediated responses promote tumorigenesis.

### 2.3. Crhr2 Deficiency Exacerbates the Severity of Intestinal Tumorigenesis in Apcmin/+ Mice, While Crhr1 Deletion Confers Tumor-Protective Effects

**Figure 3:**
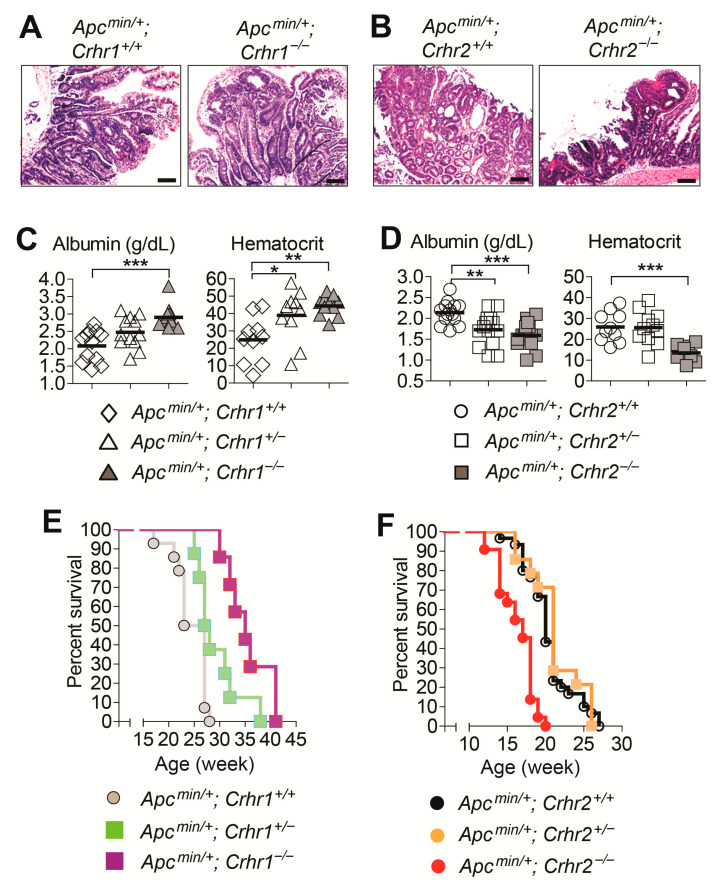
*Crhr1* deletion reduces the severity of intestinal tumorigenesis in *Apcmin/+* mice, whereas *Crhr2* deletion worsens the disease severity. (**A**,**B**) Histopathology of intestinal tumor tissues were visualized by hematoxylin & eosin (H&E) staining (scale bars, 100 μm). (**C**,**D**) Serum albumin and hematocrit levels were analyzed using mouse blood samples. *Apcmin/+; Crhr1^+/+^* (n = 10–12), *Apcmin/+; Crhr1^+/−^* (n = 10–12), *Apcmin/+; Crhr1^−/−^* (n = 11–12), *Apcmin/+; Crhr2^+/+^* (n = 10–14), *Apcmin/+; Crhr2^+/−^* (n = 11–14), *Apcmin/+; Crhr2^−/−^* mice (n = 10–15). * *p* < 0.05, ** *p* < 0.01, *** *p* < 0.001 (one-way ANOVA). (**E**,**F**) The mouse mortality was evaluated over 10 months after birth. Difference in survival was analyzed by a Kaplan–Meier plot. The log-rank (Mantel–Cox) test was used to compare significant survival difference (*p* = 0.0008 in **E**, *p* = 0.0002 in **F**). Data were analyzed with the results accumulated by 3 independent experiments. *Apcmin/+; Crhr1^+/+^* (n = 14), *Apcmin/+; Crhr1^+/−^* (n = 8), *Apcmin/+; Crhr1^−/−^* (n = 8), *Apcmin/+; Crhr2^+/+^* (n = 30), *Apcmin/+; Crhr2^+/−^* (n = 13), *Apcmin/+; Crhr2^−/−^* mice (n = 22).

With the microscopic examination of hematoxylin & eosin (H&E) stained tissue sections, we confirmed that *Apcmin/+; Crhr1^−/−^* mice and *Apcmin/+; Crhr2^−/−^* mice develop hyperplasia with enhanced mitosis. The tumor mass is characterized with massive neutrophil infiltration and marked architectural distortion of crypts with epithelial cell atypia (Figure 3A,B). These histological features were similarly observed in the tumors obtained in *Apcmin/+; Crhr1^+/+^* mice and *Apcmin/+; Crhr2^+/+^* mice. Accordingly, the histological analysis exhibited that either *Crhr1* or *Crhr2* deletion does not alter the histopathological nature of adenomas developed in *Apcmin/+* mice.

Given the massive tumor development throughout the small intestine, we next carried out blood sample analysis to evaluate the disease severity. Reduced levels of serum albumin and hematocrit indicate protein-losing enteropathy and anemia, respectively. These are atypical manifestations of severe intestinal tumorigenesis in *Apcmin/+* mice [19]. Along these lines, the level of albumin was markedly increased in *Apcmin/+; Crhr1^−/−^* mice compared to that of littermate *Apcmin/+; Crhr1^+/+^* mice (Figure 3C). Similarly, the level of hematocrit in *Apcmin/+; Crhr1^−/−^* mice was much higher than the level in *Apcmin/+; Crhr1^+/+^* mice. It is worth noting that *Apcmin/+; Crhr1^+/−^* mice had a higher level of hematocrit than *Apcmin/+; Crhr1^+/+^* mice. These data indicate that Crhr1 deficiency ameliorates the disease severity in the *Apcmin/+* mouse model of intestinal tumorigenesis.

In contrast, we observed that *Apcmin/+; Crhr2^−/−^* mice and *Apcmin/+; Crhr2^+/−^* mice have markedly reduced level of albumin compared to that of *Apcmin/+; Crhr2^+/+^* mice (Figure 3D). Similarly, reduced hematocrit level was observed in *Apcmin/+; Crhr2^−/−^* mice compared to *Apcmin/+; Crhr2^+/+^* mice. In contrast to the data from *Apcmin/+; Crhr1^−/−^* mice, this finding suggests that the *Crhr2* deficiency exacerbates the disease severity in *Apcmin/+* mice.

In line with the results from the serum analysis, survival of *Apcmin/+; Crhr1^−/−^* mice was greatly improved compared to that of *Apcmin/+; Crhr1^+/+^* mice (Figure 3E). Due to the extensive tumorigenicity, however, survival of *Apcmin/+; Crhr2^−/−^* mice was dramatically reduced compared to that of *Apcmin/+; Crhr2^+/+^* mice (Figure 3F).

Taken together, these data demonstrate that the *Crhr1* deficiency not only suppresses tumor development and growth in the *Apcmin/+* mouse, but also ameliorates the disease severity. In contrast, the *Crhr2* deficiency promotes the tumor development and worsens the disease severity.

### 2.4. Crhr1 Deficiency Reduces the Expression of Tumor-Promoting Cyclooxygenase 2 (Cox2), While Upregulating the Expression of Tumor-Suppressing Phospholipase A2 (Pla2) in Apcmin/+ Mice

The above observations prompted us to seek an explanation for how *Crhr1* gene deletion could confer a protective effect against intestinal tumorigenicity in *Apcmin/+* mice. To answer this question, we hypothesized that the Crhr1 deficiency could alter intracellular signaling networks, which contribute to suppressing the tumorigenicity. To test this hypothesis, we analyzed the expression of tumor-associated genes with adenoma tissues isolated from the mice by carrying out quantitative real-time PCR (qPCR) analyses.

**Figure 4:**
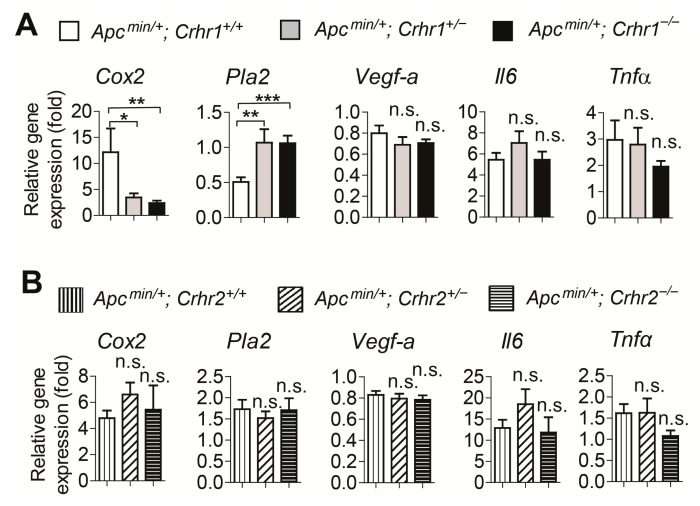
*Crhr1* deletion alters the expression of *Cox2* and *Pla2* in the tumors developed in *Apcmin/+* mice. (**A**,**B**) The mRNA levels of cancer-associated genes, including *Cox2*, *Pla2*, *Vegf-a*, *Il6*, and *Tnfα*, were evaluated by performing qPCR. Using tumor tissues isolated from the small intestine, total RNA was isolated to carry out qPCR, and the mRNA level of each gene in the tumor tissues was measured. In addition, total RNA of normal intestinal tissues from *Apcmin/+; Crhr1^+/+^* mice (**A**) or from *Apcmin/+; Crhr2^+/+^* mice (**B**) was also isolated. With qPCR, the mRNA level of each gene in the normal tissues was measured. The level of normal tissues was set as ‘1’. To this value, the mRNA level of each gene from the tumor tissues was then compared to calculate the relative gene expression in a fold unit. Presented are the accumulated data of three independent experiments. Error bars indicate ± SEM. * *p* < 0.05, ** *p* < 0.01, *** *p* < 0.001, n.s. stands for not significant (one-way ANOVA, n = 11–16 per group).

Among various genes associated with tumorigenesis, we found that the expression of *Cox2* is dramatically reduced in tumors of *Apcmin/+; Crhr1^−/−^* mice and *Apcmin/+; Crhr1^+/−^* mice, compared to the expression in adenomas from *Apcmin/+; Crhr1^+/+^* mice. It is worth noting that enhanced *Cox2* expression potently enhances both colon cancer in humans and tumorigenesis in *Apcmin/+* mice; specifically, inhibition of *Cox2* expression or its enzymatic activity by nonsteroidal anti-inflammatory drugs greatly suppresses tumor development [20]. Therefore, it is reasonable to believe that reduced *Cox2* expression by *Crhr1* deficiency may be associated with the tumor-protective effect observed in both *Apcmin/+; Crhr1^−/−^* mice and *Apcmin/+; Crhr1^+/−^* mice (Figure 4A). Furthermore, we observed that the mRNA expression of *Pla2* is markedly increased in adenomas of *Apcmin/+; Crhr1^−/−^* mice and *Apcmin/+; Crhr1^+/−^* mice, compared to the level of *Apcmin/+; Crhr1^+/+^* mice. However, the mRNA levels of tumor-associated genes, including *Vegf-a*, interleukin 6 *(Il6),* and tumor necrosis factor *α (Tnfα)*, were comparable in all adenomas from *Apcmin/+; Crhr1^−/−^, Apcmin/+; Crhr1^+/−^,* and *Apcmin/+; Crhr1^+/+^* mice. Just like the tumor protective effect elicited by reduced *Cox2* expression, it had been demonstrated that augmented *Pla2* levels are associated with suppressed tumor development in *Apcmin/+* mice [21,22]. In conjunction with reduced *Cox2* levels, our data suggested that upregulated *Pla2* expression plays a crucial role in eliciting the tumor-protective effect by *Crhr1* deficiency. However, the mRNA levels of these tumor-associated genes were similar between the adenomas from *Apcmin/+; Crhr2^−/−^, Apcmin/+; Crhr2^+/−^,* and *Apcmin/+; Crhr2^+/+^* mice (Figure 4B).

Taken together, these data indicate that a blockade of CRHR1-mediated responses should inhibit the expression of *Cox2*, and upregulate the expression of *Pla2*. Thereby, *Crhr1* gene deficiency is capable of not only suppressing the tumor development and growth, but also ameliorating the disease severity, at least in the *Apcmin/+* mouse model of intestinal tumorigenesis.

### 2.5. Crhr2 Deficiency Promotes Tumor Development and Growth in the Azoxymethane (AOM) and Dextran Sulfate Sodium (DSS)-Treated Model of Colon Cancer, While Crhr1 Deletion Does Not Alter the Tumorigenesis

**Figure 5:**
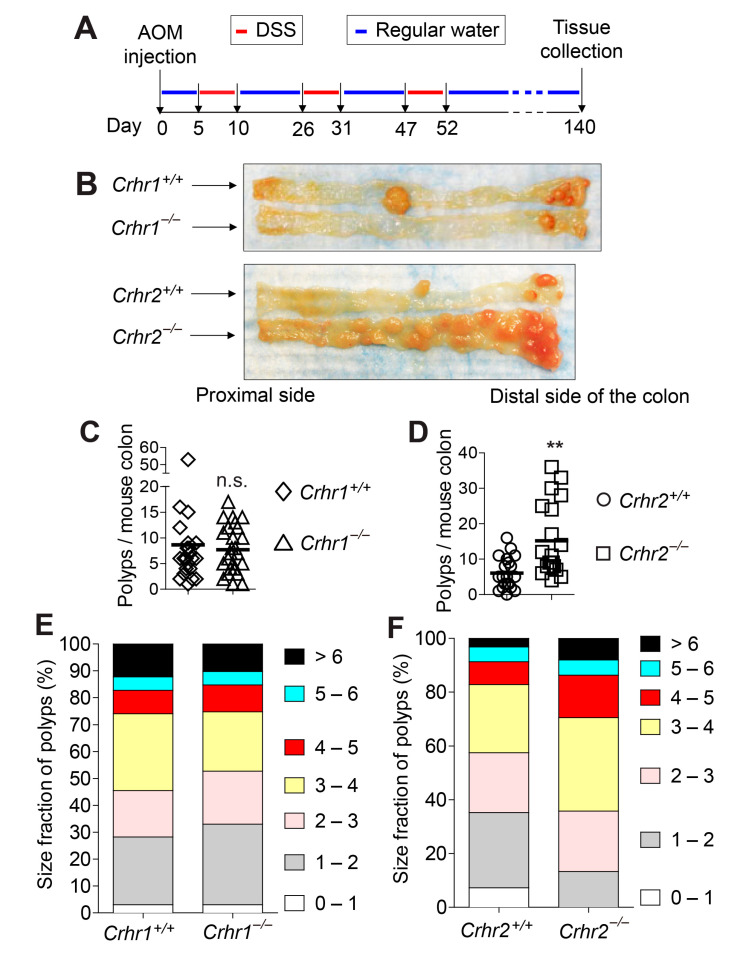
*Crhr2* deficiency enhances the tumor development and growth in the azoxymethane (AOM)/dextran sulfate sodium (DSS)-treated mouse model of colon cancer. (**A**,**B**) Presented is the experimental timeline of the AOM/DSS-treated model of colon cancer. Sex and age matched (5–6 weeks old) mice were intraperitoneally injected with AOM (12.5 mg/kg), followed by the treatment of three cycles of DSS. Then, mice were housed under normal conditions over a total of approximately 140 days before harvesting the tissues. (**B**) Gross images of tumors developed in the colon of mice treated with AOM/DSS. Tumors were mainly developed throughout the mid and distal colon. (**C**,**D**) We counted the number of visible tumors that were developed in the colon of each mouse. Horizontal bar in each graph indicates mean. ** *p* < 0.01, n.s. stands for not significant (Mann–Whitney U test). *Crhr1^+/+^* (n = 22), *Crhr1^−/−^* (n = 26), *Crhr2^+/+^* (n = 20), and *Crhr2^−/−^* (n = 20). (**E**,**F**) Tumors developed in the colon were enumerated to analyze relative proportions (%) of tumor size. Tumors were classified based on the tumor size: small (<2 mm diameter), medium-sized (2–4 mm), or large (>4 mm). Presented are the accumulated data from three independent experiments.

*Apc* is a tumor suppressor gene. In humans, somatic mutations of *Apc* result in large numbers of adenomatous polyps in the colon and rectum, which is regarded as familial adenomatous polyposis (FAP) [23]. The *Apcmin/+* mouse that harbors a heterozygous germline mutation in the Apc gene also develops multiple neoplasia in the intestine [24]. Thus, the *Apcmin/+* mouse has been widely utilized as an animal model of human colon cancer. However, *Apcmin/+* mice develop tumors predominately in the small intestine [17,18], while human FAP patients develop polyps in their colon and rectum [23]. To reconcile this discrepancy between the *Apcmin/+* mouse model and human colon cancer patients, it is necessary to examine whether CRHR1- or CRHR2-mediated responses would result in a similar effect in a different mouse model of colon cancer. Accordingly, we harnessed the AOM/DSS-induced mouse model of colon cancer, in which multiple tumors develop in the colon [25]. *Crhr1^−/−^*, *Crhr2^−/−^*, and their littermate control mice were treated with an intraperitoneal administration of AOM and DSS in drinking water to induce tumor development (Figure 5A). With this experimental approach, we observed massive tumor development primarily throughout the mid and distal colon (Figure 5B).

Subsequently, we observed that the number of polys in the colon of *Crhr1^−/−^* mice is comparable to that of *Crhr1^+/+^* mice (Figure 5C). However, the number of adenomas was dramatically increased in the colon of *Crhr2^−/−^* mice compared to that of *Crhr2^+/+^* mice (Figure 5D). These data indicate that the tumor development in the AOM/DSS model is not influenced by Crhr1 deficiency; but, *Crhr2* deficiency greatly promotes tumor development in the colon. In agreement with these data, the proportional size distribution of tumors developed in the colon of *Crhr1^−/−^* mice was similar to that of *Crhr1^+/+^* mice (Figure 5E). In contrast, the percentage proportions of large adenoma (>4 mm diameter) and medium-sized adenoma (2–4 mm) were markedly increased in *Crhr2^−/−^* mice compared to those of *Crhr1^+/+^* mice (Figure 5F).

Taken together, these data indicate that loss of the *Crhr2* gene promotes tumor formation and growth in the chemically induced model of colon cancer. In contrast to the tumor suppressing effect observed in the *Apcmin/+* mice, *Crhr1* deficiency does not alter tumorigenesis in the AOM/DSS model.

### 2.6. Crhr2 Deficiency Exacerbates the Severity of AOM/DSS-Induced Tumorigenesis

**Figure 6:**
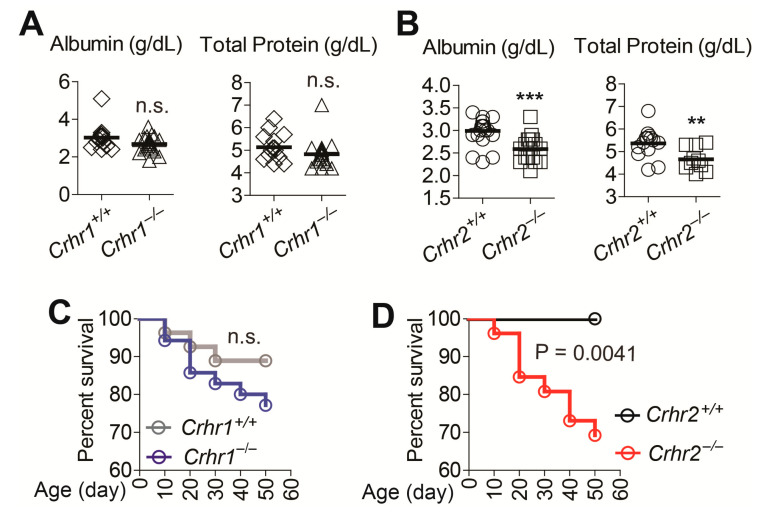
*Crhr2* deficiency exacerbates the severity of intestinal tumorigenesis in the AOM/DSS-treated mice. (**A**,**B**) Serum albumin and total protein were analyzed using mouse blood samples. Horizontal bar in each graph indicates mean. ** *p* < 0.01, *** *p* < 0.001, (Mann–Whitney U test). *Crhr1^+/+^* (n = 15), *Crhr1^−/−^* (n = 20–24), *Crhr2^+/+^* (n = 14–21), and *Crhr2^−/−^* (n = 10–17). (**C**,**D**) Difference in survival was analyzed by Kaplan–Meier plot. The log-rank (Mantel–Cox) test was used to compare significant survival difference (*p* = 0.0041 in **D**). n.s. stands for not significant. *Crhr1^+/+^* (n = 28), *Crhr1^−/−^* (n = 35), *Crhr2^+/+^* (n = 23), and *Crhr2^−/−^* (n = 26). Data were analyzed with the results accumulated by 5 independent experiments.

Next, we examine the severity of AOM/DSS-induced tumor development. The levels of serum albumin and total protein were not altered in *Crhr1^−/−^* mice compared to those of *Crhr1^+/+^* mice (Figure 6A). However, these protein levels, indicative of protein-losing enteropathy in mice with intestinal tumors, were markedly reduced in *Crhr2^−/−^* mice compared to those of *Crhr2^+/+^* mice (Figure 6B). In line with these observations, *Crhr1^−/−^* and *Crhr1^+/+^* mice had similar survival during the experimental period (Figure 6C); however, survival of *Crhr2^−/−^* mice was greatly reduced compared to that of *Crhr2^+/+^* mice throughout the experimental course of AOM/DSS-induced tumor development (Figure 6D).

Together, these data suggest that in the mouse model of AOM/DSS-induced colon cancer, *Crhr2* deficiency exacerbates tumorigenicity, while loss of *Crhr1* does not seem to alter the intestinal tumor development and growth.

## 3. Discussion

The CRH family peptides stimulate cognate CRHRs to regulate psychological stress through the HPA axis, indicating their essential roles in regulating the neuroendocrine response in the nervous system. Emerging evidence suggests an important contribution of the CRH family peptides and their receptors in regulating physiological homeostasis of peripheral organs. For instance, the expression of CRH has been observed in the adrenal gland and the gastrointestinal tract [26]. The expression of UCN I is detected in heart, skin and adipose tissue [12,27,28]. UCN II and UCN III are observed in peripheral blood cells, skeletal muscle, pancreas, and gestational tract [29,30]. Moreover, CRHR1 and CRHR2 exist in various peripheral tissues, including the heart, adrenal gland, fat tissues, skeletal muscle and skin [30,31]. Intriguingly, the expression of CRHR1 and CRHR2 is observed in the small intestine and in the colon [32,33,34]. These findings suggest that the physiological network of the CRH family system is also involved in executing physiological roles outside the HPA axis.

Nonetheless, the impact of CRHR-mediated responses in the development and growth of colon cancer clearly remains to be further investigated. In this study, we observed that the mRNA levels of *CRHR2* and its cognate ligand *UCN III* are substantially reduced in human colon cancer tissues compared to normal colon tissues (Figure 1A,B). Our finding is in agreement with the previous study that the *CRHR2* mRNA level is lower in human colon cancer tissues compared to normal tissues [34]. Together, these data strongly indicate that the CRH family peptides and their receptors should be associated with the pathogenesis or pathophysiology of colon cancer. It is of interest that in contrast to the reduced expression of *CRHR2* and *UCN III*, the expression of *UCN II* (another specific ligand of CRHR2) is markedly increased in the colon cancer tissues compared to normal tissues (Figure 1B). Given the inflammatory condition that is frequently observed within tumor tissues, we speculate that immune cells infiltrated into the tumor mass could lead to increased expression of *UCN II* in colon cancer tissues. Indeed, it has been suggested that *UCN II* expression is greatly induced in a large population of immune cells that have infiltrated into the lamina propria and submucosa of the distal colon [35].

Furthermore, to access the role of CRHR-mediated responses in colon cancer, we tested *Crhr1^−/−^* mice and *Crhr2^−/−^* mice in two different animal models of human colon cancer: the *Apcmin/+* mouse model and the AOM/DSS-treated model. In an *Apcmin/+* mouse, loss of an *Apc* allele inhibits the degradation of β-catenin, which leads to the constitutive activation of the WNT pathway to induce perpetual cell proliferation for tumor development. Therefore, *K-Ras* mutations and inactivation of *p53*, other pathways of tumor development, are not observed in the tumor development of *Apcmin/+* mice [24]. On the other hand, AOM is a carcinogen that can induce adenomas in rodents. Therefore, AOM treatment in rodents can recapitulate the initiation and progression of tumors in the colon, which is similar to the tumorigenic pathway of human colon cancer. Therefore, a combination of AOM with DSS treatment has been broadly utilized as an appropriate animal model of human colon cancer [36,37]. Due to the carcinogenic nature of AOM, the histopathology of tumors developed in the AOM/DSS model has a lot in common with human colon cancer; for instance, AOM/DSS-induced tumors have a defective mismatch repair system and mutations in *K-Ras* and β-catenin [36]. Accordingly, the AOM/DSS model is particularly useful to evaluate the activity of tumor-preventive drugs.

With these two mouse models of colon cancer, our data clearly show that *Crhr2* deficiency greatly promotes the development and growth of tumors in the intestine, while worsens disease severity in mice. Considering these data, it is reasonable to speculate that a pharmacological stimulation of the CRHR2-mediated response would induce tumor-preventive or tumor-suppressive effect in individuals with a risk of colon cancer.

In contrast, our data demonstrate that *Crhr1* deficiency markedly suppresses tumor development and growth in *Apcmin/+* mice (Figure 2 and Figure 3). However, we could not observe this tumor-suppressing effect of *Crhr1* deficiency in the AOM/DSS model of colon cancer (Figure 5 and Figure 6). Intriguingly, a study has previously showed that *Crhr1^−/−^* mice develop much fewer tumors when the mice are tested in the AOM/DSS model, suggesting that *Crhr1* deficiency can induce tumor-suppressive effects in this model [33]. In that study, the authors showed that the tumor tissues have decreased levels of inflammatory cytokines such as *Il6* and *Tnfα*. It is worth noting that the tumor development and growth in the AOM/DSS model is elicited by the degree of DSS-induced colitis; the microbiome in mice and microbial environment in an animal housing facility heavily influence the severity of DSS-induced mouse colitis. Depending on the diversity of the microflora, therefore, the tumor-suppressing effect of *Crhr1* deficiency could be recapitulated with different outcomes, providing an explanation why we could not observe the tumor-suppressing effect of *Crhr1* deficiency in the AOM/DSS model. In contrast to the AOM/DSS model that can be affected by the degree of DSS-induced colitis, our data clearly show that *Crhr1* deficiency potently inhibits tumorigenicity in the *Apcmin/+* mouse model.

Regarding the mechanism by which *Crhr1* deficiency suppresses tumorigenicity in *Apcmin/+* mice, we identified that the *Crhr1* deletion abolishes the expression of *Cox2* (Figure 4A). It is of significance that the inhibition of COX2 induces tumor-preventive effects against colon cancer [20]. In addition, *Crhr1* deficiency upregulates the level of *Pla2* in the tumor tissues. It is worth noting that PLA2 has the potential to suppress the development of colon cancer [21,22]. Accordingly, we hypothesize that *Crhr1* deficiency not only reduces the expression of *Cox2*, but also increases the *Pla2* level, thereby, suppressing tumor development and growth. Indeed, we observed that *Crhr2* deficiency does not change the expression levels of *Cox2* and *Pla2* in the tumor tissues of *Apcmin/+* mice (Figure 4B). Therefore, we speculate that unchanged expression levels of *Cox2* and *Pla2* may contribute to exacerbated tumorigenesis in *Apcmin/+; Crhr2^−/−^* mice. When it comes to the exacerbated tumorigenesis in *Crhr2^−/−^* mice tested in the AOM/DSS model, we have previously demonstrated that *Crhr2* gene deletion or CRHR2 inhibitor treatment worsens DSS-induced mouse colitis [14]. Given the pathological nature of the AOM/DSS model, it is reasonable to believe that enhanced colitis in DSS-treated *Crhr2^−/−^* mice contributes to exacerbated tumor development.

In conclusion, our data clearly suggest that the *Crhr1* deficiency confers a tumor-suppressing effect, while the *Crhr2* deficiency has the potential to worsen the development and growth of colon cancer. Therefore, either antagonists against CRHR1 or pharmacological activation of CRHR2-mediated responses should merit further investigation for developing a novel approach against colon cancer in humans.

## 4. Materials and Methods

### 4.1. Human Tissues

All human tissues were harvested and examined under the approval of the UCLA Institutional Review Board (protocol: 14-000132) and Wonkwang University Sanbon Hospital Institutional Review Board (protocol: 2011-07). Subjects (aged 32–85) who were under colonoscopy screening provided written informed consent for this study. We excluded patients with a personal or first-degree family history of cancer. Patients with previous chemotherapy or radiation therapy were excluded. Patients with any infectious disease or intestinal inflammatory disease such as IBD were also excluded. While carrying out the colonoscopy, board certified gastroenterologists harvested the colorectal cancer tissues at Wonkwang University Sanbon Hospital, South Korea. A small piece of each specimen was sent to a pathologist to determine the pathological assessment. Remaining parts of the specimen were immediately immersed in RNAlater RNA stabilization reagent (Qiagen, Valencia, CA, USA) and stored at 4 °C overnight and kept in liquid nitrogen until RNA isolation. Based on the pathologic determination, cancerous (adenocarcinoma) colonic tissues were selected for the experiment. Similarly, unmatched normal colonic mucosa specimens were collected from tumor-free healthy subjects undergoing routine colonoscopy screening at Wonkwang University Sanbon Hospital.

### 4.2. Animals

*Crhr1* knockout (*Crhr1^−/−^*) mice were obtained from the Jackson Laboratory (Bar Harbor, ME). *Crhr1^−/−^* mice and littermate *Crhr1^+/+^* mice were maintained by the breeding pairs of *Crhr1^+/−^* mice. *Crhr2*-deficient (*Crhr2^−/−^*) mice were kindly provided by Dr. W. Vale (Salk Institute, La Jolla, CA, USA) [38] and had been backcrossed to a C57BL/6 background at least more than 10 generations. Similarly, *Crhr2^−/−^* mice and littermate *Crhr2^+/+^* mice were derived from the breeding pairs of *Crhr2^+/−^* mice. *Apcmin/+* mice [39] on the C57BL/6 background were obtained from the Jackson Laboratory. Mouse genotypes were determined at the age of 3 weeks by performing genotyping PCR provided by the Jackson laboratory for *Crhr1^−/−^* and *Apcmin/+* mice and by Dr. Vale’s laboratory for *Crhr2^−/−^* mice [38]. Mice were housed under specific pathogen-free conditions in an isolator (4 mice per cage) at a constant temperature (22 °C) and in 12-h-light:dark cycle. Mice were given free access to water and unpurified mouse food (Harlan Teklad Laboratory diet #8604, Madison, WI, USA). This diet contains (g/kg): 466.4 carbohydrate, 244.8 protein, 44.0 fat, 36.9 fiber and 78.4 ash [40]. The Institutional Animal Care and Use Committee of University of California Los Angeles approved all procedures (Approval number: 2007-137; Date: 03/12/2016).

### 4.3. Apcmin/+ Mouse Model of Intestinal Tumorigenesis

We utilized the *Apcmin/+* mouse model of intestinal tumorigenesis [41,42], in which the *Apcmin/+* mouse mainly develops more polyps in the small intestine than in the colon [17,18]. *Crhr1^−/−^* and *Crhr2^−/−^* mice were crossed with *Apcmin/+* mice to generate *Apcmin/+; Crhr1^−/−^* and *Apcmin/+; Crhr2^−/−^*, respectively. During the breeding procedures, littermate control mice were also generated. These mice were maintained for 10 months after birth. During this experimental period, the mouse health conditions were monitored every other day. Large dissectible adenomas were isolated for molecular studies. After briefly staining colons in methylene blue, adenomas and aberrant crypt foci were scored under a stereoscope.

### 4.4. AOM and DSS-Treated Mouse Model of Colon Cancer

As a mouse model of colon cancer, we harnessed the AOM/DSS model [25]. To induce tumor development in the mouse colon, sex and age (5–6 weeks old) matched mice were intraperitoneally (i.p.) injected with a single dose of AOM (12.5 mg/kg), followed by normal housing for 5 days without any challenge. Then, mice were treated with two cycles of DSS (2.5%) administered in the drinking water for 5 days separated by 16 days of normal drinking water. Subsequently, mice were treated with DSS (1%) for 5 days, followed by regular drinking water treatment for 16 days. Then, mice were housed in a routine housing condition for additional 88 days. Mice were euthanized in total 20 weeks after the AOM injection. The colon tissues were harvested, longitudinally opened, and fixed for 16 h in formalin.

### 4.5. Adenoma Enumeration and Measurement of Adenoma Burden

Mice were euthanized by CO_2_ asphyxiation, and the small intestine and the colon were excised, then opened longitudinally. The tissues were rinsed with 0.9% NaCl solution, and spread onto microscope slides. The adenomas were counted, and the diameter of each adenoma was measured to the nearest 1.0 mm under an inverted light microscope with a screen at 2.5× magnification. The location and diameter of each adenoma was recorded with a millimeter scale placed on the screen. The adenomas were classified based on the tumor size: small (<2 mm diameter), medium-sized (2–4 mm), or large (>4 mm). The relative proportions of the size classes were calculated and given as a percentage of the total number of adenomas [43].

### 4.6. Clinical Assessment of Intestinal Tumorigenesis in Mice

Mice were co-housed in separate cages with less than 4 mice per cage. Mice were observed every day for morbidity and mortality. Body weight change was monitored every day during the experimental period. Kaplan–Meier survival curves were generated to analyze the mouse survival with the log-rank test [44,45].

### 4.7. Histology

The transverse colon segments (1 cm) were fixed in 10% buffered formalin, paraffin-embedded, and stained with H&E [19].

### 4.8. Quantitative Real-Time PCR (qPCR)

As described previously [44,46], total RNA was initially isolated from the tissue samples using RNeasy Plus Universal Midi Kit (Qiagen, Valencia, CA, USA). Then an equal amount of RNA (4 μg in 40 μL) was transcribed into cDNA with a High Capacity Reverse Transcription Kit obtained from “Applied Biosystems” (Foster City, CA, USA). Subsequently, quantitative real-time PCR was performed with *Taq*Man Universal Master Mix to measure gene expression by following the standard conditions from Applied Biosystems in 7500 Fast Real-Time PCR system. After incubating at 50 °C (2 min) and activating Ampli*Taq* Gold activation at 95 °C (10 min), samples were denatured at 95 °C (15 s) and annealed/extended at 60 °C (1 min) for 40 cycles. The primer pairs and FAM™ dye-labeled *Taq*Man^®^ MGB (minor groove binding) probes were purchased from Thermo Fisher Scientific (Waltham, MA, USA): *CRH* (Hs01921237_s1), *UCN2* (Hs00264218_s1), *UCN3* (Hs00846499_s1), *CRHR1* (Hs00366363_m1), *CRHR2* (Hs00266401_m1), human *GAPDH* (Hs02786624_g1), *Cox2* (Mm00478374_m1), *Pla2* (Mm01316982_m1), *Vegf-a* (Mm00437306_m1), *Il6* (Mm00446190_m1), *Tnf-alpha* (Mm00443258_m1), mouse *Gapdh* (Mm99999915_g1). Using the PCR cycle (*C*_T_) at which the probe’s fluorescent intensity passes a certain threshold value (*C*_T_) at the exponential phase, the level of expression was calculated. Through the difference in the *C*_T_ values of the target genes after normalization to RNA input level, relative gene expression was determined using *C*_T_ value of control *Gapdh*. The delta/delta *C*_T_ (2^−ΔΔ*C*^_T_) method [47] was used to calculate the relative gene expression. Each reaction was performed in triplicate.

### 4.9. Blood Sample Collection for Serum Chemistry

Blood samples were harvested by cardiac puncture from mice and collected in clot activator-containing Capillary Blood Collection Tubes, T-MG (Terumo Medical Corp., Elkton, MD, USA). Immediately after blood collection, tubes were gently inverted several times and subsequently kept upright at 4 °C for 30 min to allow for clot formation. Then, serum was separated by centrifugation at 1800× *g* for 10 min at 4 °C. The serum samples were subjected to serum chemistry analysis at the DLAM Diagnostic Laboratory at UCLA.

### 4.10. Statistical Analysis

Statistical analysis was conducted with GraphPad Prism (GraphPad Software, Inc., San Diego, CA, USA) unless stated otherwise. Additional information regarding statistical analysis is presented in the corresponding figure legend. *p* values less than 0.05 were considered significant.

## Data Availability

The data presented in this study are available on request from the corresponding author.
